# Boosting the Photoaged Skin: The Potential Role of Dietary Components

**DOI:** 10.3390/nu13051691

**Published:** 2021-05-16

**Authors:** Ruixuan Geng, Seong-Gook Kang, Kunlun Huang, Tao Tong

**Affiliations:** 1 Beijing Advanced Innovation Center for Food Nutrition and Human Health, College of Food Science and Nutritional Engineering, China Agricultural University, Beijing 100083, China; 17768128861@163.com; 2 Department of Food Engineering, Mokpo National University, Muangun 58554, Korea; sgkang@mokpo.ac.kr; 3 Key Laboratory of Safety Assessment of Genetically Modified Organism (Food Safety), Ministry of Agriculture, Beijing 100083, China

**Keywords:** extracellular matrix, fibroblast, nutraceuticals, photoaging, skin

## Abstract

Skin photoaging is mainly induced by ultraviolet (UV) irradiation and its manifestations include dry skin, coarse wrinkle, irregular pigmentation, and loss of skin elasticity. Dietary supplementation of nutraceuticals with therapeutic and preventive effects against skin photoaging has recently received increasing attention. This article aims to review the research progress in the cellular and molecular mechanisms of UV-induced skin photoaging. Subsequently, the beneficial effects of dietary components on skin photoaging are discussed. The photoaging process and the underlying mechanisms are complex. Matrix metalloproteinases, transforming growth factors, skin adipose tissue, inflammation, oxidative stress, nuclear and mitochondrial DNA, telomeres, microRNA, advanced glycation end products, the hypothalamic–pituitary–adrenal axis, and transient receptor potential cation channel V are key regulators that drive the photoaging-associated changes in skin. Meanwhile, mounting evidence from animal models and clinical trials suggests that various food-derived components attenuate the development and symptoms of skin photoaging. The major mechanisms of these dietary components to alleviate skin photoaging include the maintenance of skin moisture and extracellular matrix content, regulation of specific signaling pathways involved in the synthesis and degradation of the extracellular matrix, and antioxidant capacity. Taken together, the ingestion of food-derived functional components could be an attractive strategy to prevent skin photoaging damage.

## 1. Introduction

The skin is the largest organ of the body, and skin aging is one of the main manifestations of body aging [1]. Skin aging includes natural aging, heat aging, and photoaging [2]. Among them, photoaging is the most crucial factor causing skin aging damage. Skin photoaging is caused by long-term exposure to ultraviolet (UV) [3] and manifests as rough, dry, and sagging skin, deeper skin wrinkles, excessive skin pigmentation, or angiotelectasis [4], even leading to various benign or malignant tumors, such as solar keratosis, squamous cell carcinoma, and malignant melanoma [5]. UV activates or inhibits various signal pathways in the dermis and epidermis, leading to a decrease in the content of the extracellular matrix (ECM) and causing uneven structure or even skin collapse [6]. Methods to prevent or treat skin photoaging mainly include physical means of photoprotection (sunglasses, window films, clothing, etc.), topical treatment of active ingredients, and medical cosmetology [7]. Recently, there has been a growing awareness of the role of nutrition in skin health and specific dietary components have emerged as an effective alternative strategy to prevent and alleviate the symptoms of photoaging.

Eating well could be the best defense for our skin. There is increasing animal and clinical evidence that dietary supplementation of functional components can protect skin from photoaging damage. Phytochemicals [8], functional proteins [9] and peptides [10], functional sugars [11,12], functional oils [13], probiotics [14,15], vitamins [16,17], and minerals [18,19] are well-known to improve the photoaging-associated morphological abnormalities and functional decline.

In this review, we will summarize the advanced understanding of the molecular and cellular mechanisms of skin photoaging. Then, we furthermore intend to provide insight into the preventive and therapeutic potential of various food-derived active ingredients in skin photoaging and their underlying mechanisms.

## 2. Materials and Methods

Research articles that focused on mechanisms of UV-induced skin photoaging and nutraceutical intervention for skin photoaging were collected from various search engines such as Pubmed, Google Scholar, Scopus, and Science Direct using keywords including skin, photoaging, extracellular matrix, fibroblast, nutraceuticals, etc. The studies identified were reviewed and relevant citations within these studies were also reviewed.

## 3. Skin Architecture

The skin is the first defense barrier of organisms, covering the whole body, and it is the largest organ, accounting for around 16% of body weight. The area of an adult’s skin is around 1.2–2.0 m^2^. Skin is in direct contact with the external environment and has the functions of feeling external stimuli, regulating body temperature, excreting skin metabolites, and protecting the body from physical, mechanical, and chemical damage and invasion by pathogenic microorganisms. Skin consists of three parts: stratified epidermis, dermis, and subcutaneous tissue [20]. The epidermis is composed of keratinocytes (90–95% of skin cells), Langerhans cells (2%), melanocytes (3%), and Merkel cells (0.5%) [21]. The epidermis contains the stratum corneum, hyaline layer, granular layer, and germinal layer, from the shallowest to the deepest. The stratum corneum is the key to maintaining optimal skin hydration. Cells in the germinal layer continue to proliferate and migrate to the upper layer to supply the constantly shedding stratum corneum. The germinal layer contains melanocytes that can produce melanin [22] and the content of melanin is one of the factors that determines skin color.

The dermis is made up of connective tissue and also contains appendages including sweat glands, sebaceous glands, blood vessels, and nerves [23]. The dermis is divided into the papillary layer and reticular layer, and there is no obvious boundary between the two layers. The thickness of the dermis is around 0.07–0.12 mm; the dermis of the palms and soles is thicker (~1.4 mm); the eyelids and tympanic membrane are thinner (~0.05 mm). The papillary layer is connected to the germinal layer of the epidermis, and the reticular layer is combined with the subcutaneous tissue [24].

One of the main cell types in the dermis is fibroblasts, which play a vital role in skin aging [25]. Fibroblasts synthesize and secrete ECM, including collagen, hyaluronic acid (HA), and elastin [26,27]. Collagen is the most important ECM in the dermis of the skin. Loss of collagen will directly lead to skin sagging, aging, and decreased elasticity. HA is synthesized at the plasma membrane by HA synthases 1–3 and is known to play a key role in wound healing and tissue repair processes due to its ability to maintain a humid environment [28]. In the dermal fibroblasts, HA synthase 2 seems to be the major isoform [29]. Elastin is the main component of elastic fibers in matrix tissue and provides resilience and elasticity to tissues and organs. The reticular layer contains collagen fibers, elastic fibers, and reticular fibers, which interweave into a net to create elasticity and toughness in the skin [9]. Existing evidence suggests that damage to macromolecules present in the dermal ECM is indeed associated with skin aging [30].

The subcutaneous tissue is composed of loose connective tissue and fat lobules, and it connects the dermis with the fascia, aponeurosis, or periosteum. It can buffer mechanical pressure, store energy, and maintain body temperature [31].

## 4. UV-Induced Skin Photoaging

Sunlight is primarily composed of 53% infrared light, 44% visible light, and 3% UV light. UV is electromagnetic radiation with a wavelength of 100–400 nm. According to the different radiation wavelength, UV can be divided into long-wave UV (UVA, with a wavelength of 315–400 nm), medium-wave UV (UVB, with a wavelength of 280–315 nm), and short-wave UV (UVC, the wavelength is 200–280 nm).

A moderate amount of UV radiation can kill microorganisms, regulate the nerves, endocrine, digestion, breathing, immune system, and promote the synthesis of vitamin D. However, exposure to chronic low-dose or instant high-dose UV radiation causes harm to the eyes, skin, and immune system and is associated with the clinical hallmarks of skin aging. Photoaging is mainly caused by UVA and UVB. UVA has a strong ability to induce free radicals and lipid peroxidation in cells and undermines collagen fibers and elastic fibers in the dermal tissue. The impact of UVA can reach the deep layer of the dermis due to its greater penetration ability. Although UVA has no direct effect on DNA damage, it can generate reactive oxygen species (ROS), leading to DNA oxidative damage indirectly. In contrast, UVB chiefly leads to lesions in the epidermis and superficial dermis and could be absorbed by proteins and DNA in cells, causing cell damage and mutation.

Studies have shown that more than 80% of facial skin aging is caused by exposure to UV [4]. The macroscopic characteristics of skin photoaging include wrinkle formation, rough texture, pigmentation, and loss of skin elasticity. Histological and ultrastructural studies have shown epidermal hyperplasia, damage, and disorder of collagen fibers, and a large accumulation of abnormal elastic substances in connective tissue in photoaged skin [32,33]. These effects are less pronounced in the epidermis owing to high turnover. In contrast, the dermal region is more susceptible to photodamage, which results in loss of skin resilience [34].

Experimental models used for photoaging research mainly contain animal models, cell models, and 3D skin models. In addition to SKH-1 or HR-1 hairless mice, BALB/c mutant hairless mice and normal mice with shaved backs are also suitable for skin research. Epidermal HaCaT cells, dermal Hs68 cells, and dermal CDL-986sk cells are the most commonly applied cell models, while 3D skin models are not widely used in research due to their high price and complicated operation.

## 5. Mechanisms of Skin Photoaging

Skin photoaging is a complex process. In recent years, the mechanisms underlying skin photoaging have been intensively studied. Multifaceted signaling pathways and molecules are found to play important regulatory roles in this process. In the following section, we summarize the current knowledge about mechanisms of photoaging.

### 5.1. Matrix Metalloproteinases (MMPs)

MMPs are a group of endopeptidases that depend on metal ions such as Ca^2+^ or Zn^2+^, and they are secreted by keratinocytes and dermal fibroblasts under various stimuli such as oxidative stress and UV radiation [35]. MMPs is critical in skin photoaging and can degrade almost all ECMs, such as collagen, fibronectin, elastin, and proteoglycan [36]. MMPs have a similar structure and are generally composed of five structural domains, including a hydrophobic signal peptide sequence, a propeptide region that keeps the zymogen stable, a catalytically active region containing a binding site, a proline-rich hinge region, and a carboxy-terminal region related to the substrate specificity of enzymes. Among them, the catalytically active region and propeptide region are highly conserved. Different MMPs have their own characteristics. One MMP can degrade multiple ECMs, and one ECM can be broken by various MMPs.

Twenty-eight members of the human MMP family have been isolated and identified [36]. They can be divided into five subgroups: (1) Collagenase (MMP-1, MMP-8, and MMP-13) recognizes the substrate through the hemophilia protein-like domain and degrades collagen; (2) Gelatinase (MMP-2 and MMP-9) can digest a variety of ECMs, such as type I collagen and type IV collagen; (3) Stromelysins (MMP-3, MMP-10, and MMP-11) are similar to collagenase, but cannot cut type I collagen; (4) Stromelysins (MMP-7 and MMP-26) lack hemophilin-like domains and degrade type IV collagen but not type I collagen; (5) The membrane-type MMPs (MMP-14, MMP-15, and MMP-16) break down type I collagen. MMPs in mice are similar to humans, and studies also found the high expression and significant role of MMPs in mouse skin [37].

Studies have shown that UV radiation activates MMPs and induces their expression in skin [38]. Among MMPs, MMP-1, MMP-3, and MMP-9 play major roles in skin photoaging. MMP-1 can degrade type I and type III collagen, almost destroying collagen completely; MMP-3 extensively cuts type IV collagen, proteoglycans, and fibronectin; MMP-9 further degrades the collagen fragments produced by MMP-1 [36]. MMPs reduce ECMs, resulting in skin aging.

**Figure 1:**
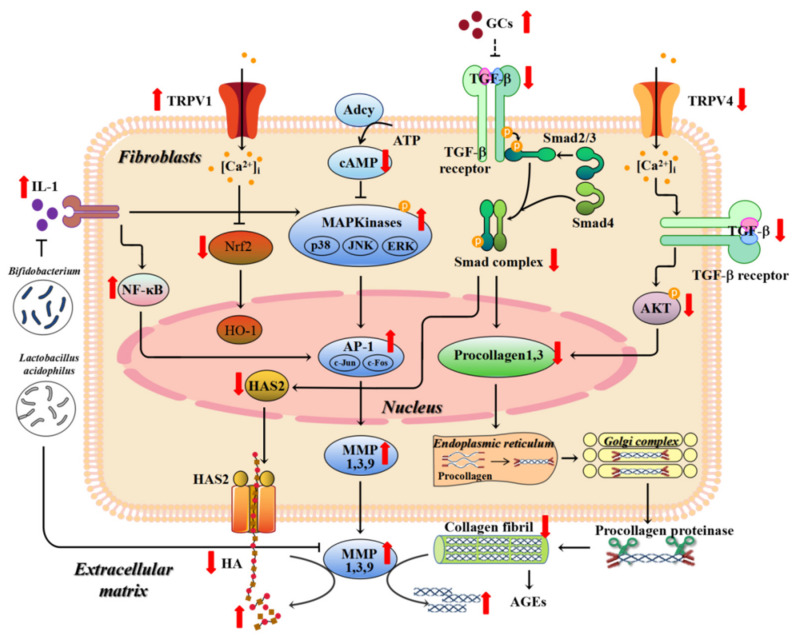
The mechanisms by which UV irradiation regulates collagen and hyaluronic acid content in dermal fibroblasts. Adcy: adenylyl cyclase; AGEs: advanced glycation end products; AP-1: activating protein-1; ERK: extracellular signal-regulated kinase; GC: glucocorticoid; HA: hyaluronic acid; HAS2: hyaluronic acid synthase 2; HO-1: heme oxygenase 1; IL: interleukin; JNK: Jun N-terminal kinase; MAPK: mitogen-activated protein kinase; MMP: matrix metalloproteinase; NF-κB: nuclear factor-κB; Nrf2: nuclear factor-E2-related factor 2; TGF: transforming growth factor; TRPV: transient receptor potential cation channel V.

Fibroblasts contribute to MMP production by UV-induced activating protein-1 (AP-1) activation (Figure 1). Briefly, UV radiation induces excessive production of ROS, and ROS excite the mitogen-activated protein kinase (MAPK) family. MAPK is composed of extracellular signal-regulated kinase, p38, and c-Jun N-terminal kinase. c-Jun combines with c-Fos to form transcription factor AP-1, which is critical in the regulation of MMP-1, MMP-3, and MMP-9 [39]. Studies have shown that cAMP (cyclic adenosine monophosphate), the important “second messenger”, regulates MMP expression by affecting the MAPK/AP-1 pathway, leading to collagen degradation [40]. In addition to AP-1, nuclear factor-κB (NF-κB) is another vital transcription factor in photoaging. The generation of ROS induces the activation of NF-κB, which is related to the upregulation of MMP-1 and MMP-3 in dermal fibroblasts [39].

### 5.2. TGF (Transforming Growth Factor)-β

TGF-β is a type of cytokine that regulates cell growth and differentiation and performs a central role in the inflammatory response, tissue repair, embryonic development, and the immune response. In canonical TGF-β signaling, TGF-β binds to two types of serine and/or threonine kinase receptors, TGF-βR (TGF-β receptor) I and TGF-βRII, triggering intracellular signaling cascades. When TGF-β binds to the TGF-βRII/TGF-βRI complex, drosophila mothers against decapentaplegic protein (Smad) 2 and Smad3 are phosphorylated by TGF-βRI, followed by binding with Smad4 to form a heteromeric complex, and translocate to the nucleus, where they exert transcriptional regulation of TGF-β target genes including multiple collagens through Smad-binding elements [41]. Moreover, TGF-β is known to downregulate ECM-degrading MMPs and to upregulate plasminogen activator inhibitor 1 and tissue inhibitor of metalloproteases, which inhibit MMP activation [42]. UV radiation decreases type I procollagen production mainly by inhibiting the TGF-β/Smad signaling pathway [43]. UVB light transcriptionally downregulates the TGF-β receptor and disrupts the downstream TGF-β–Smad signaling required to biosynthesize procollagen (Figure 1).

Crosstalk of the TGF-β/Smad pathway with several other pathways has been reported, although experimental evidence to explain such crosstalk is limited. The UV radiation activates the MAPK/AP-1 pathway, and AP-1 is known to inhibit procollagen gene expression by blocking TGF-β type II receptor–Smad signaling [42,44]. The TGF-β/Smad pathway is also affected by the cAMP/protein kinase A pathway, causing changes in collagen content [45]. In addition, the activation of the TGF-β pathway potently stimulates hyaluronan synthesis via upregulation of HA synthase 2 (Figure 1). This stimulatory effect requires the kinase active TGF-β1 receptor and is dependent on Smad signaling and activation of the p38 [46].

### 5.3. Reduction of Skin Adipose Tissue

Skin-associated adipose tissue includes dermal and subcutaneous adipocytes. Both dermal white adipose tissue (DWAT) [47] and subcutaneous white adipose tissue (SWAT) [48] play crucial roles in skin photoaging.

DWAT is a unique layer of adipocytes within the reticular dermis of the skin [47]. Human dermal adipose cells can protrude into the upper dermis and create a “fat bridge” between the skin surface and the subcutaneous fat, connecting the area directly irradiated by UV with the deeper fat layer [49]. The replacement rate of dermal adipose cells is much higher than that in SWAT. Long-term excessive exposure to UV leads to DWAT depletion and skin fibrosis at the same time. The reason for this phenomenon may be adipocyte–myofibroblast transition [50]. Substituting fibrosis for DWAT volume results in uneven skin structure and causes skin folds.

UV radiation also has a significant effect on SWAT metabolism. The amounts of free fatty acid and triglycerides in the SWAT of the photoaged forearm (sun-exposed skin) were significantly less than those in the buttocks (sun-protected skin) of the same elderly individuals [48]. Chronic UV radiation dramatically inhibits the differentiation of preadipocytes and the expression of peroxisome proliferator activated receptor γ, reducing the accumulation of triglycerides in mature adipocytes [51]. The reason might be that some soluble factors, such as IL-6, IL-8, and monocyte chemotactic protein-3, diffuse into SWAT under UV radiation and transform the metabolism of SWAT [52]. The reduction of SWAT leads to the thinning and collapse of connective tissue, which is manifested as skin atrophy and wrinkle formation.

### 5.4. Inflammation and Immune Disorders

Under UV radiation, AP-1 and NF-κB are activated, leading to the synthesis of nitric oxide synthase and cyclooxygenase. These inflammatory enzymes cause the secretion of pro-inflammatory cytokines, including IL-1β, IL-6, IL-8, and tumor necrosis factor-α, in normal human dermal fibroblast cells [53]. IL-1 could activate the MAPK pathway under UV irradiation, eventually increasing MMP-1 in human skin fibroblasts [54]. Furthermore, UV radiation induces keratinocytes to release various cytokines that influence the immune balance in skin. Three cytokines, IL-10, IL-12, and interferon-γ, have been widely studied. IL-10 appears to have an immunosuppressive function, such as inhibiting the antigen-presentation ability of the Langerhans cells and suppressing the contact hypersensitivity response [55]. On the other hand, IL-12 and interferon-γ have immunopotentiating effects that promote and enhance T helper cell 1 activity [56].

### 5.5. Oxidative Stress

ROS, such as superoxide ions, H_2_O_2_, and hydroxyl radicals, are chemically active and oxidize unsaturated fatty acids from cell phospholipid molecules to malondialdehyde, directly hurting functional macromolecules such as biofilms and proteins [57]. UV radiation induces the production of ROS, which breaks the dynamic balance between oxidation and antioxidant systems in the skin [58] and reduces its ability to remove ROS. Under this condition, ROS increase and accumulate in the body, causing the abnormal activation of signaling pathways such as NF-κB, affecting mitochondrial membrane potential, and inducing mitochondrial DNA (mtDNA) damage and cell apoptosis. Furthermore, ROS can also activate the MAPK/AP-1 pathway and stimulate the expression of MMPs, promoting the degradation of collagen and resulting in skin photoaging [59].

Iron-catalyzed ROS generation has been intensively studied in numerous pathological conditions, including skin photoaging. Under normal conditions, the iron is sequestered by iron-binding proteins, such as transferrin and ferritin, and it does not participate in ROS generation [60]. Nevertheless, UV irradiation induces an increase in the level of iron in the skin tissues. For example, Bissett et al. demonstrated the deposition of free iron in the dermis in a histological study of UVB-irradiated hairless mouse skin [61]. Smith et al. reported that UVA irradiation causes immediate increases in cytosolic and mitochondrial Fe^2+^ in human dermal fibroblasts [62]. Through the Fenton and Haber Weiss reactions, the free iron potentiates the generation of highly reactive oxygen free radicals such as hydroxyl radical, thus resulting in oxidative damage and skin photoaging [63]. Moreover, the released iron also induces direct oxidation of the biomolecules via an equilibrium between the ferrous ions and ferric [64].

### 5.6. Nuclear DNA and mtDNA Damage

UV radiation can directly or indirectly hurt DNA [65]. UVA radiation forms a large amount of ROS and causes nuclear DNA damage indirectly; this type of damage is mainly repaired by base-excision repair. UVB can directly injure cell DNA and generate mutation, and this DNA damage is restored by the nucleotide excision repair system. When UV radiation breaks the balance of DNA repair, it will arouse cell apoptosis, skin aging, wrinkles, and even cancer. In addition, DNA damage-driven epigenetic change is also a reason for skin aging [66].

Mitochondrion is the main organelle involved in skin photoaging [67]. The loss and mutation of mtDNA are common phenomena in skin aging [68]. As there is a plethora of evidence to support the close connection between mitochondria and skin health, the strategy of focusing on mitochondria as a therapeutic target to boost skin health has attracted the attention of clinicians and estheticians. A great quantity of bioactive compounds have been confirmed to ameliorate mitochondrial function and have positive effects on aging and diseased skin [69].

### 5.7. Telomere Shortening

Telomeres are critical structures at the end of eukaryotic chromosomes made up of numerous copies of G-rich repeats. Without telomeres, chromosomes will fuse and genetic instability will occur [70]. Telomerase, the tightly regulated enzyme complex that maintains telomere length in rapidly proliferating cells such as germline and cancer cells, has been found to play a key role in the maintenance of skin cell function and proliferation [71]. UV radiation is known to induce excessive ROS production, resulting in telomere mutations and further cell death [72]. While some initial findings point to the likely possibility that telomerase dysfunction and/or telomere shortening in skin fibroblasts and keratinocytes are important for the aging process in skin [73], further studies are clearly required.

### 5.8. MicroRNA (miRNA)

In the natural aging process, miRNA 217 regulates the senescence of human skin fibroblasts by directly targeting DNA methyltransferase 1 [74]. MiR–23a–3p controls cellular senescence by targeting enzymes to control HA synthesis in human fibroblasts [75]. Moreover, UVB alters miR-34 family expression in mouse dorsal skin, in addition to dysregulating collagen structure, with subsequent reductions in strength and elasticity [76]. These results suggest that miRNAs may play a pivotal role in regulating ECM deposition and skin biomechanics following chronic UVB exposure, and thus may be a possible target for therapeutic development. Further in vivo and in vitro investigations are warranted to decipher the exact role of these miRNA in the photoaging process.

### 5.9. Accumulation of Advanced Glycation End Products (AGEs)

A prominent feature of aging at the molecular level is the gradual accumulation of proteins that have undergone non-enzymatic modification, one of the commonest of which is glycation. Reducing sugars react with free amino groups on proteins (and other molecules), leading to the reversible production of reactive intermediates and ultimately to irreversible AGEs. Long-lived proteins in the dermal matrix and cytoskeleton are particularly susceptible to glycation, resulting in tissue stiffening and reduced elasticity [77]. Among extracellular proteins, glycated elastin fibers abnormally aggregate and unusually interact with lysozymes in the skin of the solar elastosis but not sun-protected sites, indicating that glycation is involved in photoaging [78].

### 5.10. Gut Microbes

The gut and skin, densely vascularized and richly innervated organs with crucial immune and neuroendocrine roles, are uniquely related in purpose and function [79]. As our primary interface with the external environment, both organs are essential to the maintenance of physiologic homeostasis.

The gut microbiome is the major regulator of the gut–skin axis. Oral probiotics may counteract UV damage [80] and relieve inflammatory dermatoses [81] by regulating immune-related signal pathways. Gut dysbiosis has been observed in conditions such as atopic dermatitis [82]. The mechanisms by which intestinal microbiota exert their influence on skin homeostasis appear to be related to the modulatory effect of gut microbes on systemic immunity [79]. Further mechanistic studies will be required to understand the role of gut microbes in the process of skin photoaging.

### 5.11. Activation of Hypothalamic–Pituitary–Adrenal (HPA) Axis

The HPA axis is associated with the activation of a wide range of responses involving the endocrine, nervous, and immune systems, collectively known as the stress responses [83]. Skin has neuroendocrine capabilities that also encompass all elements of the HPA axis [84]. Mechanistic studies of mouse skin in vivo demonstrate that UVB can upregulate the expression of all the elements of the HPA axis, including corticotrophin-releasing hormone, proopiomelanocortin, β-endorphin, melanocortin receptor 2, steroidogenic enzymes, and glucocorticoid (corticosterone) [85].

The primary actions of glucocorticoids are mediated by the glucocorticoid receptor, a transcription factor that regulates many complex signaling pathways. Long-term topical and systemic use of glucocorticoids is associated with skin atrophy, disruption to cutaneous barrier function, and dermatitis [86]. Although it is unclear how glucocorticoid application results in these phenotype changes, potential mechanisms may involve the inhibition of the TGF-β signal. For instance, methylprednisolone ameliorates early cardiac dysfunction after coronary microembolization and suppresses TGF-β1/Smad3 expression [87]; cortisol inhibits the activity of TGF-β1 in osteoblasts from fetal rats [88]; in the tissue repair of the dermis, dexamethsone restrains the TGF-β signal and collagen synthesis [89].

### 5.12. Transient Receptor Potential Cation Channel V (TRPV)

The TRP ion channel is a type of channel protein widely distributed in the peripheral and central nervous system. TRP allows cations to pass through the cell membrane non-selectively and is responsible for various sensory responses, including heat, cold, pain, stress, vision, and taste. The TRP family now includes more than 30 cation channels [2], and TRPV is one of the main subfamilies of TRP. The TRPV family plays vital roles in the process of skin photoaging. The opening of TRPV triggers Ca^2+^ influx, activates different signaling pathways, causes ECM changes and inflammatory reactions, and results in skin photoaging eventually.

In human dermal fibroblasts, UVB irradiation causes a Ca^2+^ increase via TRPV1, induces nuclear factor-E2-related factor 2 (Nrf2) degradation, leads to an imbalance of intracellular redox homeostasis, and brings about skin photoaging finally [90] (Figure 1). In HaCaT cells, UV irradiation activates Src kinase, which induces TRPV1 trafficking from intracellular vesicles to the cell membrane [91]. Moreover, activation of TRPV1 also mediates the expression of MMP-1 in natural-aging and heat-aging skin [2]. The activation of the TRPV3 channels regulates the dermal structure by reducing ECM production via the TRPV3/Thymic stromal lymphopoietin/Smad2/3 pathways in dermal fibroblasts [92]. In the process of photoaging, it is worth exploring whether the TGF-β pathway regulates TRPV3. Inhibiting the expression of TRPV4 can attenuate photoaging damage of epidermal cells and mouse skin, indicating the potential of TRPV4 as a therapeutic target for photoaging [93]. However, some research reports that the activation of TRPV4 has a positive effect on the formation and recovery of the epidermal barrier [94]. Furthermore, TRPV4 can activate the TGF-β1/AKT signal and enhance the differentiation of dermal myofibroblasts and the increase in collagen content induced by TGF-β1 [95] (Figure 1). Nevertheless, the lack of animal tests raises questions about the true effectiveness of these TRPVs in vivo, highlighting the need for more studies in living systems to examine the exact role of TRPVs in the process of photoaging.

## 6. Efficacy and Mechanisms of Dietary Components in Mitigating Skin Photoaging: Animal and Human Evidence

Nowadays, with the improvement of living standards and the deepening of health concepts, boosting photoaged skin through diet has attracted more and more attention. In this section, we will summarize the existing scientific evidence from mouse (Table 1) and human in vivo studies (Table 2) that supports the beneficial impacts of oral administration of dietary components on skin photoaging.

### 6.1. Phytochemicals

#### 6.1.1. Carotenoids

Astaxanthin has diverse functions in skin biology, including photoprotective, antioxidant, and anti-inflammatory effects [96]. Oral administration of astaxanthin is protective against UV-induced skin deterioration and is helpful to maintain healthy skin. In a 10-week, double-blind, placebo-controlled study, there was reduced loss of back skin moisture and the back skin texture was significantly improved in participants who were supplemented with astaxanthin, compared with a placebo group [97,98].

Lycopene is a tetraterpene compound abundantly found in tomato and tomato-based products and is recognized as a potent antioxidant. Lycopene has been found to be efficient in skin photoaging. Participants ingested 55 g tomato paste (16 mg lycopene) in olive oil or olive oil alone daily in a 12-week, randomized controlled trial. The tomato paste group showed reduced expression of MMP-1 and mtDNA damage in the upper buttock skin, suggesting that oral administration of tomato paste containing lycopene provides protection against photodamage [99].

The combined consumption of lycopene and other carotenoids also attenuates skin photoaging. For example, oral supplementation with lycopene and lutein promotes human skin health. In a 12-week, double-blinded, placebo-controlled, crossover study, the capacity of lycopene-rich tomato nutrient complex and lutein was examined at a molecular level. The experimental results showed that oral supplementation of lycopene and lutein completely inhibited the upregulation of heme oxygenase-1, intercellular adhesion molecule 1, and MMP-1 mRNA in arm skin [100].

Oral administration of a nutritional supplement containing lycopene, β-carotene, and *Lactobacillus johnsonii* prevents skin damage from polymorphic light eruption. In a 12-week, randomized, placebo-controlled, double-blinded study, intake of the supplement significantly reduced the polymorphic light eruption score after UVA exposure as compared with patients taking a placebo. At a molecular level, the development of skin lesions was associated with increased expression of intercellular adhesion molecule 1 mRNA [101].

Protection from UV radiation seems more effective upon treatment with combined tomato antioxidant compounds, compared to the effects of lycopene treatment alone. For example, a study showed that the protective effect of a tomato extract containing lycopene, phytofluene, and phytoene against UV-induced erythema formation was more pronounced compared with lycopene alone [102]. This could be associated with the fact that the interaction between structurally different antioxidant molecules may provide more comprehensive protection against oxidative injury [103].

#### 6.1.2. Polyphenols

Immature *Citrus unshiu* is known to contain high concentrations of flavonoids such as hesperidin and narirutin. Oral administration of immature *Citrus unshiu* powder improves UVB-induced loss of skin hydration, the increase in transepidermal water loss, and the overgrowth of epidermal cells, while suppressing epidermal cell mortality and basement membrane destruction, in hairless mice [104].

Oral supplementation with hydrangenol mitigated wrinkle formation, dorsal thickness, dehydration, and collagen degradation in HR-1 hairless mice. Hydrangenol increased the expression of Pro-COL1A1 and HA and upregulated the expression of Nrf2, oxygenase-1, NAD(P)H quinone dehydrogenase 1, glutamate cysteine ligase modifier subunit, and glutamate cysteine ligase catalysis subunit in mouse dorsal skin. Hydrangenol attenuated the phosphorylation of MAPKs and reduced the expression of MMP-1/-3, cyclooxygenase-2, and IL-6 in mouse dorsal skin [105].

Dihydromyricetin, a flavonoid, and ellagic acid, a polyphenol dilactone, both found in fruits and vegetables, are used for anti-photoaging treatment. Oral supplementation with a combination of dihydromyricetin and ellagic acid had a synergistically protective action against UVB damage in the dorsal skin tissue of mice, manifested as significantly ameliorated erythema, and markedly reduced the expression of pro-inflammatory cytokines and MMP-1, compared with the separate treatment of dihydromyricetin and ellagic acid. Their beneficial effects may be associated with the activation of both TGF-β1 and wnt signaling [106].

Chang et al. showed that oral administration of hawthorn polyphenol extract protects against UVB-induced facial skin photoaging in female Balb/c mice. Hawthorn polyphenol extract reversed epidermal thickening and dermal damage and promoted the production of type I procollagen in the dorsal skin of UVB-irradiated mice through the inactivation of NF-κB and the phosphorylation of MAPK. In addition, dietary supplementation with hawthorn polyphenol extract decreased the production of ROS and increased the antioxidant enzyme activity in mouse dorsal skin [107]. This group later demonstrated that oral treatment with hawthorn polyphenol extract also inhibits skin photoaging through the p53 mitochondrial pathway [108].

Resveratrol is a naturally occurring polyphenolic phytoalexin found in grapes, red wine, peanuts, mulberries, and fruits [109]. In a placebo-controlled, double-blind clinical study, facial skin moisturization and elasticity were enhanced, while facial skin roughness and depth of wrinkles were reduced, in subjects who were orally supplied with a resveratrol–procyanidin blend. Additionally, plasmatic antioxidant capacity and skin antioxidant power increased significantly [110]. Similarly, a clinical trial demonstrated the protective effect of curcumin, a polyphenol compound isolated from turmeric, on skin photoaging and inflammation. In this randomized, double-blind, placebo-controlled trial, oral supplementation with curcumin improved the moisture content of facial skin and inhibited increases in UVB-induced tumor necrosis factor-α and IL-1 in skin [111]. Given the reported anti-photoaging effect of curcumin and the fact that curcumin acts as an iron chelator [112], it is likely that curcumin may eventually improve skin photoaging by removing iron ions in the skin and reducing skin oxidative damage; nevertheless, future confirmatory studies are needed.

Green tea catechin, a natural iron chelator and antioxidant [113], might scavenge hydroxyl radicals and provide protection against UV-induced skin damage. Indeed, oral supplementation with green tea polyphenols containing catechin, epicatechin, epigallocatechingallate, epicatechingallate, epigallocatechin, and glucuronidase/sulfatase protects against the UV-induced sunburn response, immunosuppression, and photoaging of the skin. In a 12-week, double-blind, placebo-controlled study, supplementation with green tea polyphenols significantly reduced the UV-induced erythema in facial skin, improved skin elasticity, roughness, density, and water homeostasis, and increased the blood flow and oxygen delivery to the skin [114].

A study investigated the efficacy of a combination of rosemary (*Rosmarinus officinalis*) and grapefruit (*Citrus paradisi*) polyphenols in decreasing the individual susceptibility to UV exposure. This randomized, parallel-group study showed that oral administration of dietary polyphenols reduced the UV-induced facial skin redness and lipoperoxides and improved facial skin wrinkles and elasticity [115].

#### 6.1.3. Plant Extracts and Fermentation

Cacao beans are known to contain a variety of bioactive compounds and their consumption is associated with skin health. Oral administration of cacao powder was able to attenuate UVB-induced mouse dorsal skin wrinkling by the reduction of MMP-1 via the MAPK pathway and the downregulation of the gene expression of cathepsin G in SKH-1 mice and a 3D skin model [116].

Oral administration of mycosporine-like amino acids extracted from *Porphyra tenera* had a protective effect against UV irradiation-induced photoaging by activating the NF-kB pathway in mouse skin. Mycosporine-like amino acids significantly inhibited the decrease in hydroxyproline and collagen content, improved pathological damage to collagen fibers, and reduced the expression of MMP-1, MMP-3, and tumor necrosis factor-α in mouse dorsal skin [117].

Garlic has been reported to exert positive effects on the skin structure and to protect the skin from the damages arising from UV exposure. Oral administration of garlic diminished UV-induced coarse wrinkle formation and ameliorated mouse dorsal skin and epidermal thickness. The expression of procollagen mRNA was increased in the dorsal skin of the garlic-supplied mice, while MMP-1 and MMP-2 protein and mRNA levels were reduced. In addition, oral supplementation of garlic significantly decreased ROS generation and mouse dorsal skin and serum malondialdehyde levels, and upregulated superoxide dismutase and catalase activities in mouse dorsal skin tissue [118].

Oral supplementation with dietary *Foeniculum vulgare Mill* extract attenuated UVB irradiation-induced skin photoaging by activating Nrf2 and inhibiting MAPK pathways in HR-1 mice. This extract dramatically improved the production of collagen, elastin, and TGF-β1 in mouse dorsal skin, while reducing skin MMP levels under UVB irradiation [119].

Wheat extract oil supplementation improved UVB-induced losses in skin moisture, elasticity, and damage of skin barrier function, and inhibited the decreases in procollagen type I, HA, and ceramide, in mouse dorsal skin tissue [120].

Oral supplementation with a fermentation of blackberry with *L. plantarum JBMI F5* protects the skin from UVB-induced photoaging through regulation of MAPK/NF-κB signaling. Fermented blackberry diminished wrinkle formation and epidermal thickening in mouse dorsal skin, maintained ECM density, and reversed type-1 procollagen reduction and antioxidant enzyme inactivation [121].

### 6.2. Proteins and Peptides, Carbohydrates, and Fattty Acids

#### 6.2.1. Proteins and Peptides

The application of collagen and its hydrolysate in skin aging has received growing attention [9]. Oral administration of collagen hydrolysate from silver carp (*Hypophthalmichthys molitrix*) skin ameliorated skin photoaging in mice. The ingestion of collagen hydrolysate led to a dose-dependent increase in the skin content of hydroxyproline, HA, and moisture. Moreover, ingesting collagen hydrolysate with lower (200–1000 Da, 65%) and higher molecular weight (>1000 Da, 72%) markedly improved the antioxidative enzyme activities in both serum and skin of Kunming mice [10].

In a double-blind, placebo-controlled study, the effectiveness of an oral supplement of the specific bioactive collagen peptide VERISOL^®^ on eye skin was assessed. Eye wrinkle formation was reduced, and biosynthesis of procollagen I, elastin, and fibrillin in the skin was significantly increased in the bioactive collagen peptide-treated group [122].

Asserin et al. evaluated the effect of oral administration of collagen peptide supplementation on facial skin moisture and the dermal collagen network. Oral administration of collagen peptide (a specific mixture of collagen peptides of fish origin or porcine origin) significantly increased skin hydration and collagen density in facial skin and upregulated glycosaminoglycan production in ex vivo experiments [123].

Another study showed that oral supplementation with collagen hydrolysate and antioxidant-containing nutraceuticals significantly decreased wrinkle width, open pores, skin roughness, and the color of hyperpigmented blemishes, and enhanced skin hydration, firmness, elasticity, and barrier function, in female participants’ eye skin [124].

#### 6.2.2. Carbohydrates

Plant polysaccharides have attracted increasing attention for their anti-photoaging effects. For example, both *Tremella fuciformis* and *Sargassum fusiforme* polysaccharides enhance the activity of antioxidant enzymes, such as superoxide dismutase and catalase, reduce oxidative stress, and ultimately improve skin damage. Oral supplementation with *Tremella fuciformis* polysaccharides reduced UV-induced water and collagen losses in skin, repaired collagen breakdown, maintained a stable type I/III collagen ratio, and increased the levels of glycosaminoglycans in the dorsal skin of Sprague-Dawley rats. Furthermore, after treatment with *Tremella fuciformis* polysaccharides, the activities of superoxide dismutase, catalase, and glutathione peroxidase were improved in dorsal skin [11]. Oral administration of *Sargassum fusiforme* polysaccharide protected hairless Kunming mice from UVB radiation by inhibiting MMP-1 and MMP-9 in skin. Moreover, *Sargassum fusiforme* polysaccharide enhanced superoxide dismutase and catalase activities, and decreased ROS and malondialdehyde levels, in mouse dorsal skin, resulting in the reduction of UVB-induced oxidative stress [12].

Galacto-oligosaccharide is a non-artificially synthesized oligosaccharide derived from animal milk and is an excellent prebiotic with bifidobacteria proliferation activity. In a randomized, double-blind clinical trial, oral treatment with galacto-oligosaccharides reduced the transepidermal water loss and facial wrinkle area [125].

HA is a widely available polysaccharide and plays a vital role in skin structure [126]. Clinical studies have proven that oral supplementation with HA could improve skin moisture and elasticity and relieve wrinkles and roughness in eye skin [127].

#### 6.2.3. Fatty Acids

Oral supplementation with 7-MEGA^TM^ 500, an unsaturated fatty acid, could alleviate UVB-induced photoaging in hairless mouse skin. Oral treatment with 7-MEGA™ 500 improved skin thickness, skin barrier function, and wrinkle indicators, and reduced the expression of MMP-3 and c-Jun, in mouse dorsal skin [128].

Supplementation with dietary suberic acid, a dibasic fatty acid, protected against UVB-induced skin photoaging in hairless mice. Oral administration of suberic acid restrained UVB-induced skin dryness, wrinkle formation, and epidermal thickness. Mechanistically, suberic acid upregulates the expression of molecules in the TGF-β/Smad pathway and inactivates the MAPK/AP-1 pathway. Oral suberic acid downregulated the expression of MMP-1, MMP-3, and MMP-9 and enhanced the expression of collagen and HA in the back skin of mice [129].

Administration of olive oil promoted collagen synthesis, decreased catecholamine synthesis, and inhibited ROS production, lipid peroxidation, protein carbonylation, and MMP-8 levels in stressed mouse dorsal skin [13]. Squalene, a polyunsaturated aliphatic hydrocarbon found in shark liver oil and olive oil, is an important intermediate in the endogenous synthesis of cholesterol [130]. In a clinical study, oral supplementation with squalene substantially increased procollagen levels and reduced facial wrinkles and erythema, keratinocytic apoptosis, and thymine dimer levels in facial skin [131].

Oral administration of fish oil may offer a protective effect against skin photoaging. Research suggests that fish oil supplementation (containing eicosapentaenoic acid and docosahexaenoic acid) increases minimal erythema dose significantly and decreases erythema and p53 induction in human skin [132]. Although fish oil intake reduces UV-induced damage, the lipid peroxidation of skin increases due to the unstable nature of n-3 fatty acids [133].

Oral supplementation with argan oil leads to a significant improvement in skin health. In a clinical research, oral administration of argan oil increased the gross elasticity, net elasticity, and biological elasticity in volar forearm skin, and decreased resonance running time markedly, in postmenopausal women [134].

Tanaka et al. demonstrated that oral intake of plant sterols of aloe vera gel (Aloe sterol) improved the skin condition in the photoaged skin of both men and women. In a 12-week, randomized, double-blind, placebo-controlled study, oral supplementation with aloe sterol increased skin elasticity in the photodamaged skin of men in the inner side of the forearm [135]. The group later demonstrated that oral supplementation with aloe sterol also significantly increased skin elasticity, hydration, and the collagen score in women’s inner forearm skin [136].

#### 6.2.4. Other Animal-Derived Active Substances

There is accumulating evidence that insect consumption can alleviate photoaging and improve skin health. Oral supplementation with four insect extracts including *Allomyrina dichotoma* larva, *Protaetia brevitarsis seulensis*, *Tenebrio molitor* Linnaeus, and *Gryllus bimaculatus* De Geer relieved skin winkles, epidermal thickening, barrier dysfunction, loss of transepidermal water, and collagen breakdown and inhibited the expression of MMPs, phosphorylation of MAPK, and relief of pro-inflammatory cytokines. Furthermore, the level of skin hydration-related markers including HA, TGF-β, and procollagen was upregulated markedly through treatment with insect extracts after UVB exposure [137].

### 6.3. Probiotics

Probiotics are defined as viable microorganisms that exert a beneficial effect on the health of the host when they are ingested in sufficient quantity. Mounting evidence suggests that probiotics may play a significant role in skin health via regulating intestinal microbiota and metabolites and improving systemic immunity [79,138,139,140]. This section introduces several probiotics that are beneficial to skin and their mechanisms.

Oral supplementation with *bifidobacterium breve B-3* improved skin photoaging induced by chronic UV irradiation in mice. B. breve B-3 markedly relieved transepidermal water loss and epidermal thickening, suppressed the damage to the tight junction structure and basement membrane, and attenuated the production of IL-1β in skin [15].

Administration of tyndalized *Lactobacillus acidophilus* can significantly enhance the recovery ability of photoaged skin. Mice administered with tyndalized *Lactobacillus acidophilus* displayed a significant reduction in wrinkle formation and transepidermal water loss, and an increase in skin hydration. Oral administration of tyndalized *Lactobacillus acidophilus* suppressed the expression levels of MMP-1 and MMP-9 in skin [141].

Lactic acid bacteria play an essential role in the food industry in the manufacture of many fermented products, and the application of lactic acid bacteria is now being extended to the area of health improvement. Oral supplementation with *lactococcus lactis H61* improves the status of inner forearm and cheek skin in Japanese women by adjusting the balance of intestinal microbes, exerting antioxidant activity, and regulating the immune response [14].

Oral supplementation with *lactobacillus paracasei NCC 2461* promotes the recovery of skin health. A randomized, double-blind, placebo-controlled clinical trial showed that *lactobacillus paracasei NCC 2461* decreased skin sensitivity through inhibition of the release of the neuromediators involved in sensitivity reaction, and improved skin barrier function in leg and cheek skin [142].

Compared with the above-mentioned phytochemicals, collagen peptides, and plant polysaccharides, knowledge of the mechanisms of probiotics is lacking and unclear, although probiotics show promising photoprotective effects. It will be of great interest to delineate the underlying anti-photoaging mechanisms of probiotics in further studies.

### 6.4. Vitamins and Minerals

Vitamins are crucial to delay skin aging and improve skin appearance. Vitamin A is a fat-soluble vitamin and was the first vitamin approved by the US Food and Drug Administration as an anti-wrinkle agent. Vitamin A and its derivatives are some of the most effective substances to relieve aging [143]. Clinical studies have shown that oral supplementation with vitamin A and its derivatives can alleviate skin photoaging. Histological and immunohistochemical analysis revealed elastosis reduction, epidermal thickness increase, corneal layer diminution, dermal collagen 1 increase, and epidermal p53 reduction in the facial and forearm skin of 50- to 75-year-old men and women [17].

Vitamin C is a strongly water-soluble antioxidant. The skin contains a high concentration of vitamin C, which is much higher than the plasma concentration [141]. Vitamin C is the key to collagen biosynthesis and can directly promote the expression of collagen genes [144]. It also protects against photoaging by removing ROS generated by UV radiation. Vitamin C acts as a cofactor for the proline and lysine hydroxylases, and proline and lysine hydroxylase are essential in the process of micelle formation and in stabilizing the collagen molecule tertiary structure [145]. The dependence of collagen hydroxylase on vitamin C has been confirmed in many in vitro studies [144,146]. Oral supplementation with vitamin C could prevent dry skin, reduce wrinkle formation, and promote wound healing [147].

Vitamin E is a fat-soluble vitamin and a strong antioxidant. In the last century, clinical studies have shown that oral vitamin C and E can relieve sunburn on human back skin. A double-blind, placebo-controlled study demonstrated that supplementation with a combination of vitamins C (2 g/day) and E (1000 IU/day) reduced the sunburn reaction [148]. Similarly, Fuchs and Kern demonstrated that dietary supplementation with megadoses of vitamin E (2 g/day) combined with vitamin C (3 g/day) protected participants from the sunburn reaction [149]. The reason might be that the combination of vitamin C and E can efficaciously eliminate water-soluble and fat-soluble free radicals at the same time, preventing free radicals from causing oxidative damage to the cell membrane. The doses of vitamin C and E used in the above-mentioned trials are higher than the corresponding recommended dietary allowance. The adverse effects of vitamin C, even at doses of 10 to 20 g/day, have not been convincingly demonstrated [150]. Few side effects from vitamin E have been reported. Doses up to 400 mg can be considered absolutely safe, and vitamin E doses between 400 and 2000 mg also seem to cause no side effects [151,152].

In addition, minerals such as copper, zinc, and selenium also play important roles in skin health [19,153,154]. They are cofactors in enzymatic reactions during collagen crosslinking and necessary elements for epidermal proliferation and keratinocyte differentiation [154,155].

## 7. Conclusions and Perspectives

Skin photoaging has gradually become an injury that cannot be underestimated. Significant progress has been made in the understanding of mechanisms of skin photoaging. MMPs, TGFs, reduction in skin adipose tissue, inflammation and immune disorders, oxidative stress, nuclear DNA and mtDNA damage, telomere shortening, miRNA, accumulation of AGEs, gut microbes, activation of the HPA axis, and TRPV are the main mechanisms causing photoaging. UV radiation reaches skin and triggers multiple mechanisms and pathways at the same time. These mechanisms seem not to be independent of each other and can connect through intermediate signaling molecules. In the future, it is necessary to identify new target molecules of skin photoaging and explore the mutual regulation among existing mechanisms.

Diet is key to skin health. A number of clinical and preclinical studies have demonstrated that dietary supplementation of phytochemicals, collagen, functional sugars, functional oils, probiotics, and vitamins plays an essential role in anti-photoaging efficacy. These functional ingredients have been reported to promote skin health and prevent skin photoaging through the activation of different signaling pathways. Future research is needed to (i) examine the anti-photoaging action and dose of these dietary components in clinical trials and identify the most promising functional ingredient candidates for humans, as the potentiality of most dietary components has been examined only in preclinical trials, either in vitro or in vivo; (ii) explore more active ingredients with protective ability against photoaging, through animal models, 3D skin models, and cell models; (iii) identify metabolite products of the dietary intake of functional ingredients through novel methods including skin metabolomics, as metabolites might be effective to relieve skin photoaging; and (iv) study the bioavailability of functional ingredients and their effects on whole body metabolism. These will contribute to a more detailed comprehension of the photoaging-preventive effects of dietary components, boosting the development of functional ingredients as a tool to counteract skin photoaging.

## Figures and Tables

**Table 1:** Summary of dietary components to boost photoaged skin in animal experiments.

Ingredients	Model	Dose	Duration	Main Results	Reference
Phytochemicals					
Immature *Citrus unshiu*	HR-1 hairless mice, male, 6 weeks old	200 mg/kg body weight/day	7 weeks	skin hydration ↑ transepidermal water loss ↓ overgrowth of epidermal cell ↓ epidermal cell mortality ↓ basement membrane destruction ↓	[104]
Hydrangenol	HR-1 hairless mice, male, 5 weeks old	5, 10, 20, 40 mg/kg/day	7 weeks	collagen, HA ↑ Nrf2, HO-1 ↑ wrinkles, dorsal thickness, dehydration ↓ HAS-1/-2 ↓ MAPKs, MMP-1/-3 ↓ COX-2 ↓ IL-6 ↓	[105]
Dihydromyricetin and ellagic acid	ICR mice, male, 6 weeks old	0.7% cellulose; 0.7% ellagic acid; 0.7% dihydromyricetin or 0.35% ellagic acid and 0.35% dihydromyricetin	3 weeks	TGF-β1 ↑ wnt ↑	[106]
Hawthorn polyphenol	BALB/c mice, female, 5–6 weeks old	100, 300 mg/kg/day	12 weeks	antioxidant enzyme activity ↑ type I procollagen ↑ epidermal thickening, dermal damage ↓ ROS ↓ MAPKs ↓ NF-kB ↓	[107]
Hawthorn polyphenol	BALB/c mice, female, 5–6 weeks old	100, 300 mg/kg/day	12 weeks	ROS ↑ DNA damage ↓ p53 activation ↓ caspase activation ↓	[108]
Cocoa extract	SKH-1 hairless mice, female, 6 weeks old	39.1, 156.3, 625 mg/kg	8 weeks	MAPK, MMP-1 ↓ cathepsin G ↓ wrinkles ↓	[116]
Mycosporine-like amino acids extracted from *Porphyra tenera*	ICR mice, male	5, 10, 20 mg/mL	30 days	NF-kB ↑ hydroxyproline ↑ collagen ↑ MMP-1, MMP-3 ↓ TNF-α ↓	[117]
Garlic supplementation	SKH-1 hairless mice, female, 6 weeks old	1%, 2%	8 weeks	dorsal skin, epidermal thickness ↑ procollagen ↑ SOD, CAT ↑ wrinkles ↓ ROS ↓ MDA ↓ MMP-1, MMP-2 ↓	[118]
*Foeniculum vulgare mill* extract	HR-1 mice, male, 7 weeks old	0.1%, 1%	10 weeks	collagen ↑ Nrf2 ↑ elastin ↑ TGF-β 1 ↑ MAPK, MMPs ↓	[119]
Wheat extract oil	SKH-1 hairless mice, 6 weeks old	30, 60, 120 mg/kg	12 weeks	moisture, skin elasticity ↑ procollagen type I, HA ↑ ceramide ↑	[120]
Fermentation of blackberry with *L. plantarum JBMI F5*	SKH-1 hairless mice, female, 6 weeks old	158 mg/kg of blackberry and 1 × 10^10^ CFU of *L. plantarum JBMI F5*	4 weeks	type-1 procollagen ↑ antioxidant enzyme ↑ ECM density ↑ MAPK/NF-κ B signaling ↓ wrinkles ↓ epidermal thickening ↓	[121]
Functional proteins and peptides, sugars, or oils					
Collagen hydrolysates from silver carp (*Hypophthalmichthys molitrix*) skin	Kunming mice, female, 5 weeks old	50, 100 and 200 mg per kg body weight collagen hydrolysates	6 weeks	hydroxyproline ↑ HA ↑ moisture contents ↑ antioxidative enzyme activities ↑	[10]
*Tremella * *fuciformis* polysaccharides	SD rats, female, 6/7 weeks old	100, 200, 300 mg/kg/day	4 weeks	moisture contents ↑ collagen ↑ stability of type I/III collagen ratio ↑ glycosaminoglycans ↑ SOD, CAT ↑	[11]
*Sargassum fusiforme* polysaccharide	Kunming mice, female, 7 weeks old	200, 400, 600 mg/kg/day	9 weeks	SOD, CAT ↑ MMP-1, MMP-9 ↓ ROS, MDA ↓ oxidative stress ↓	[12]
7 mega™500	HR-1 hairless mice, male, 5 weeks old	50, 100, 200 mg/kg	12 weeks	wrinkles ↓ MMP-3 ↓ c-Jun ↓	[128]
Dietary suberic acid	SKH-1 hairless mice, female, 6 weeks old	0.05%, 0.1%, 0.2% suberic acid	10 weeks	TGF-β/smad pathway ↑ COL1A1, COL1A2, COL3A1 ↑ HAS1, HAS2, HAS3 ↑ skin dryness ↓ wrinkles ↓ epidermal thickness ↓ MAPK/AP-1 pathway ↓ MMP-1a, MMP-1b, MMP-3, MMP-9 ↓	[129]
Olive oil	Swiss mice, male, 8–12 weeks old	1.5 g/kg per day, contained 74.7 g of oleic acid cis 9 (C18:1) per 100 g of oil and 0.104 mg/mL of total polyphenols	4 weeks	collagen ↑ ROS ↑ lipid peroxidation ↓ protein carbonylation ↓ MMP-8 ↓	[13]
Insect extracts	HR-1 hairless mice, male	0.1 mL extracts containing 100 mg/kg body weight	12 weeks	Collagen, HA ↑ TGF-β ↑ winkles ↓ epidermal thickness ↓ barrier dysfunction ↓ loss of transepidermal water ↓ MAPK, MMPs ↓ pro-inflammatory cytokines ↓	[137]
Probiotics					
*Bifidobacterium breve B-3*	HR-1 hairless mice, male, 6 weeks old	2 × 10^9^ cfu/mouse /day	7 weeks	tight junction structure, basement membrane ↑ transepidermal water loss ↓ epidermal thickness ↓ IL-1β ↓	[15]
Tyndalized *Lactobacillus acidophilus*	HR-1 hairless mice, male, 6 weeks old	100 mg tyndalized *Lactobacillus acidophilus*/kg body weight/day	12 weeks	skin hydration ↑ transepidermal water loss ↓ MMP-1, MMP-9 ↓ wrinkles ↓	[141]

Abbreviations: CAT, catalase; COX, cyclooxygenase; ECM, extracellular matrix; HA, hyaluronic acid; HAS, hyaluronic acid synthase; HO-1, heme oxygenase-1; IL, interleukin; MAPK, mitogen-activated protein kinase; MDA, malondialdehyde; MMP, matrix metalloproteinase; NF-kB, nuclear factor-κB; Nrf2, nuclear factor-E2-related factor 2; ROS, reactive oxygen species; SOD, superoxide dismutase; TGF, transforming growth factor; TNF, tumor necrosis factor.

**Table 2:** Summary of dietary components to boost photoaged skin health in clinical trials.

Ingredients	Country	Sample Subjects and Size	Study Design	Dose	Duration	Main Results	Reference
Phytochemicals							
Astaxanthin	Japan	Human, 23	a randomized, double-blind, placebo-controlled trial	4 mg	10 weeks	skin moisture ↑ skin texture ↑	[97]
Lycopene	UK	Women, 20, mean age 33 years	a randomized controlled study	55 g tomato paste (16 mg lycopene)	12 weeks	MED ↑ MMP-1 ↓ mtDNA ↓	[99]
Lycopene and lutein	Germany	Human, 65	a double-blinded, placebo-controlled, crossover study	5 mg lycopene and 10 mg lutein	12 weeks	HO-1 ↓ intercellular adhesion molecule 1 ↓ *MMP-1* ↓	[99]
Lycopene, β-carotene and *Lactobacillus johnsonii*	France	PLE patients, 17 males and 43 females, 60	a randomized, placebo-controlled, double-blinded study	nutritional supplement containing 2.5 mg lycopene, 4.7 mg of β-carotene and 5 × 10^8^ cfu of the probiotic *Lactobacillus johnsonii*	12 weeks	*ICAM1* ↑ PLE score ↓	[101]
Resveratrol– procyanidin blend	Italy	Men and women, 50, aged 35–65 years	a placebo-controlled, double-blind study	8 mg transresveratrol and 14.63 mg procyanidin	60 days	skin moisturization, elasticity ↑ values for systemic oxidative stress, plasmatic antioxidant capacity, skin antioxidant power ↑ skin roughness, wrinkles ↓	[110]
Green tea polyphenols	Germany	Women, 60, aged 40–65 years	a double-blind, placebo-controlled study	1402 mg green tea polyphenols	12 weeks	skin elasticity, roughness ↓ water homeostasis ↑ blood flow and oxygen delivery to skin ↑ erythema ↓	[114]
Curcumin	Japan	Human, 47	a randomized, double-blind, placebo-controlled trial	30 mg curcumin	8 weeks	water content ↑ TNF-α ↓ IL-1 ↓	[111]
Rosemary (*Rosmarinus officinalis*) and grapefruit (*Citrus paradisi*) polyphenols	Spain	Women, 90	a randomized, parallel-group study	Long-term: 250 mg/day; Short-term: 100, 250 mg/day	Long-term: 2 weeks; Short-term: 24, 48 h	skin redness ↓ wrinkles ↓ skin elasticity ↑	[115]
Functional proteins and peptides, sugars, or oils							
Bioactive collagen peptide VERISOL^®^	Brazil	Women, 114, aged 45–65 years	a double-blind, placebo-controlled study	2.5 g	8 weeks	procollagen I, elastin, fibrillin ↑ wrinkles ↓	[122]
collagen peptide	Japan	Women, 33, aged 40–59 years	an ex vivo model and randomized, placebo-controlled clinical trial	10 g specific mixture of collagen peptides of fish origin (Peptan^®^F) or porcineorigin (Peptan^®^P)	12 weeks	skin hydration ↑ collagen density ↑ glycosaminoglycan ↑	[123]
A collagen hydrolysate and antioxidant- containing nutraceutical	India	Women, 34, mean age 39.5 years	-	Marine collagen peptides (5 g of fish collagen peptides) and antioxidant blend (natural tomato extract, grape seed extract, green tea extract, vitamin C and vitamin E)	30 days	skin hydration, firmness, elasticity, barrier function ↑ wrinkle width, open pores, skin roughness, the color of hyperpigmented blemishes ↓	[124]
Galacto-oligosaccharides	Korea	Women	a randomized, double-blind clinical trial	1 g twice a day	12 weeks	TEWL ↓ wrinkles ↓	[125]
Squalene	Korea	Women, 40, >50 years	-	13.5, 27 g/day	90 days	procollagen ↑ MED ↑ facial erythema ↓ keratinocytic apoptosis ↓ thymine dimer level ↓ wrinkles ↓	[131]
Fish oil	US	Human, 10	-	10 capsules per day of fish oil containing each 280 mg EPA and 120 mg DHA	4 weeks	MED ↑	[132]
Argan oil	Morocco	Postmenopausal women, 60	-	25 mL/day	60 days	gross elasticity of skin, net elasticity of skin, biological elasticity ↑ resonance running time ↓	[134]
Aloe sterol	Japan	Men, 48	a randomized, double-blind, placebo-controlled study	40 mg/d	12 weeks	skin elasticity ↑	[135]
Aloe sterol	Japan	Women, 64	a randomized, double-blind, placebo-controlled study	aloe sterol yogurt contained 40 µg of aloe sterol per 100 g	12 weeks	skin elasticity ↑ skin hydration ↑ collagen ↑	[135]
Probiotics							
*Lactococcus H61*	Japan	Women, 30	a double-blind, placebo-controlled trial	4 × 10^10^ cfu	8 weeks	balance of intestinal microbes and intestinal health ↑ antioxidant activity ↑ skin immune response ↑	[14]
*Lactobacillus paracasei NCC 2461*	France	Women, 64, aged 18–40 years	a randomized, double-blind, placebo-controlled study	1 × 10^10^ cfu	4 weeks	barrier function ↑ skin sensitivity ↓	[142]

Abbreviations: HO-1, heme oxygenase-1; ICAM, intercellular adhesion molecule; IL, interleukin; MED, minimal erythema dose; MMP, matrix metalloproteinase; mtDNA, mitochondrial DNA; PLE, polymorphic light eruption; TEWL, transepidermal water loss; TGF, transforming growth factor.

## Data Availability

Not applicable.
